# Deptor protects against myocardial ischemia-reperfusion injury by regulating the mTOR signaling and autophagy

**DOI:** 10.1038/s41420-024-02263-1

**Published:** 2024-12-19

**Authors:** Qunjun Duan, Weijun Yang, Xian Zhu, Zhanzeng Feng, Jiangwei Song, Xiaobin Xu, Minjian Kong, Jiayan Mao, Jian Shen, Yuqin Deng, Rujia Tao, Hongfei Xu, Wei Chen, Weidong Li, Aiqiang Dong, Jie Han

**Affiliations:** 1 Department of Cardiovascular Surgery, The Second Affiliated Hospital of Zhejiang University School of Medicine, Hangzhou, Zhejiang China; 2 Key Laboratory of Cancer Prevention and Therapy Combining Traditional Chinese and Western Medicine of Zhejiang Province, Cancer Institute of Integrated Traditional Chinese and Western Medicine, Zhejiang Academy of Traditional Chinese Medicine, Tongde Hospital of Zhejiang Province, Hangzhou, Zhejiang China; 3 Department of Cardiothoracic Surgery, The First Affiliated Hospital, School of Medicine, Zhejiang University, Hangzhou, Zhejiang China; 4 Department of Cardiology and Atrial Fibrillation Center, The First Affiliated Hospital, Zhejiang University School of Medicine, Hangzhou, Zhejiang China

**Keywords:** Cell biology, Molecular biology

## Abstract

Deptor knockout mice were constructed by crossing Deptor Floxp3 mice with myh6 Cre mice, establishing a myocardial ischemia-reperfusion (I/R) model. Deptor knockout mice exhibited significantly increased myocardial infarction size and increased myocardial apoptosis in vivo. ELISA analysis indicated that the expression of CK-MB, LDH, and CtnT/I was significantly higher in the Deptor knockout mice. Deptor siRNA significantly reduced cell activity and increased myocardial apoptosis after I/R-induced in vitro. Deptor siRNA also significantly up-regulated the expression of p-mTOR, p-4EBP1, and p62, and down-regulated the expression of LC3 after I/R induction. Immunofluorescence indicated that LC3 dual fluorescence was weakened by Deptor knockout, and was enhanced after transfection with Deptor-overexpression plasmids. Treatment with OSI027, a co-inhibitor of mTORC1 and mTORC2, in either Deptor knockout mice or Deptor knockout H9C2 cells, resulted in a significant reduction in infarction size and apoptotic cardiomyocytes. ELISA analysis also showed that the expression of CK-MB, LDH, and CtnT/I were significantly down-regulated by treatment with OSI027. CCK-8 cell viability indicated that cell viability was enhanced, and the number of apoptotic cells was decreased in vitro following treatment with OSI027. These results revealed that OSI027 exerts a protective effect on myocardial ischemia/reperfusion injury in both an in vivo and in an in vitro model of I/R. These findings demonstrate that Deptor protects against I/R-induced myocardial injury by inhibiting the mTOR pathway and by increasing autophagy.

## Introduction

Acute myocardial infarction (AMI), commonly referred to as a heart attack, is a myocardial necrosis event caused by unstable ischemic syndrome, is characterized by myocardial necrosis caused by acute ischemia or sustained ischemia and hypoxia, and is the most serious cardiovascular disease with the highest morbidity and mortality worldwide [1, 2]. AMI caused by acute ischemic attack can result in massive myocardial cell death, and reperfusion therapy for AMI may lead to more severe myocardial dysfunction after ischemia [3]. Although emergency coronary revascularization restores blood supply to ischemic myocardium, tissue damage to the myocardium is progressively worse after reperfusion, a phenomenon known as myocardial ischemia-reperfusion (I/R) injury [4]. Myocardial I/R injury expands the infarction area, leads to the aggregation of a large amount of inflammatory cells in the original ischemic myocardium, seriously damages vascular endothelial function, and leads to metabolic dysfunction, arrhythmia, and apoptosis of myocardial cell, thereby aggravating AMI [5]. Reducing myocardial injury caused by myocardial I/R may significantly reduce postoperative complications and lower mortality rates, and may ultimately improve survival for those who experience myocardial infarction.

Myocardial cell death induced by myocardial I/R plays a central role in the progression of AMI, mainly through apoptosis, necrosis, and autophagic death [6]. Autophagy, which normally serves to maintain cardiac structure and function, is activated in response to stress, and limits cardiac damage under most conditions. Abnormal autophagy is related to a variety of pathological states, including cancer, neurodegenerative diseases, and cardiovascular disease [7]. Autophagy in cardiomyocytes plays an important role in cardiovascular disease and is a major regulator of cardiac homeostasis and function [8]. Induction of autophagy protects against myocardial I/R-induced cell death by inhibiting cardiomyocyte apoptosis in a myocardial I/R model [9, 10]. The role of mTOR, a key regulator of autophagy, is also important in myocardial I/R [11, 12]. MTOR is an important downstream target of the AMPK and phosphatidylinositol 3-kinase (PI3K)/Akt pathways, is essential for protein synthesis, regulates autophagy during ischemia, and is considered to be a primary regulatory mechanism of autophagy [13]. Therefore, developing new methods to regulate the mTOR pathway could provide novel and effective treatments for myocardial I/R injury.

The DEP-domain containing mTOR-interacting protein (Deptor), a newly discovered mTOR-binding protein, is encoded by a gene located on chromosome 8 (8Q24.12) and is highly homologous between different organisms [14]. Deptor consists of two N-terminal tandem Dep domains and one C-terminal PDZ domain [15]. The PDZ domain of Deptor mediates the interaction between Deptor and mTOR and inhibits the activity of mTORC1 and mTORC2 [16]. Downregulation of Deptor results in increased mTOR activity, which in turn leads to a further decrease in Deptor expression. Inhibition of Deptor also activates the mTORC1 and mTORC2 signaling pathways and negatively regulates autophagy by inhibiting the Atg1 kinase complex [17]. The protein mTORC1 regulates different steps in the process of autophagy [18], and is a potential target for regulating autophagy. Indeed, mTORC1 affects autophagosome biogenesis by phosphorylation and inactivating the autophagy regulatory complex formed; under nutrient-rich conditions, mTORC1 is phosphorylated by mediating autophagy activating kinase 1 (ULK1) and Atg13 at specific sites, which in turn inhibits the autophagy-promoting kinase activity of the ULK1 complex; during starvation and cellular stress, mTORC1 activity is inhibited and is separated from ULK1 activity [18–20]. Furthermore, Deptor interacts with mTOR to maintain activation of class III PI3K and AKT, and reduces apoptosis [21]. In addition, Deptor, as a tumor suppressor downstream of the PI3K/AKT/mTOR signaling pathway, inhibits AKT-induced protein synthesis, cell proliferation, and AKT pathway activation by blocking the mTOR signaling pathway [22, 23]. However, under certain conditions, Deptor may act as an oncogene involved in the feedback inhibition of PI3K by S6K1, thereby activating mTORC2/AKT, and contributing to AKT activation in multiple myeloma and triple-negative breast cancer, which is associated with drug resistance and tumor metastasis [24, 25]. However, the role of Deptor in myocardial I/R injury remains undetermined.

In the present study, we constructed Deptor conditional knockout mice as a model for myocardial I/R injury. We analyzed the effects of Deptor on the mTOR pathway and cardiomyocyte autophagy during myocardial I/R injury. This study provides a novel justification to pursue Deptor as a treatment for myocardial I/R injury.

## Results

### Construction of myocardial cell-specific Deptor knockout mice

**Fig. 1:**
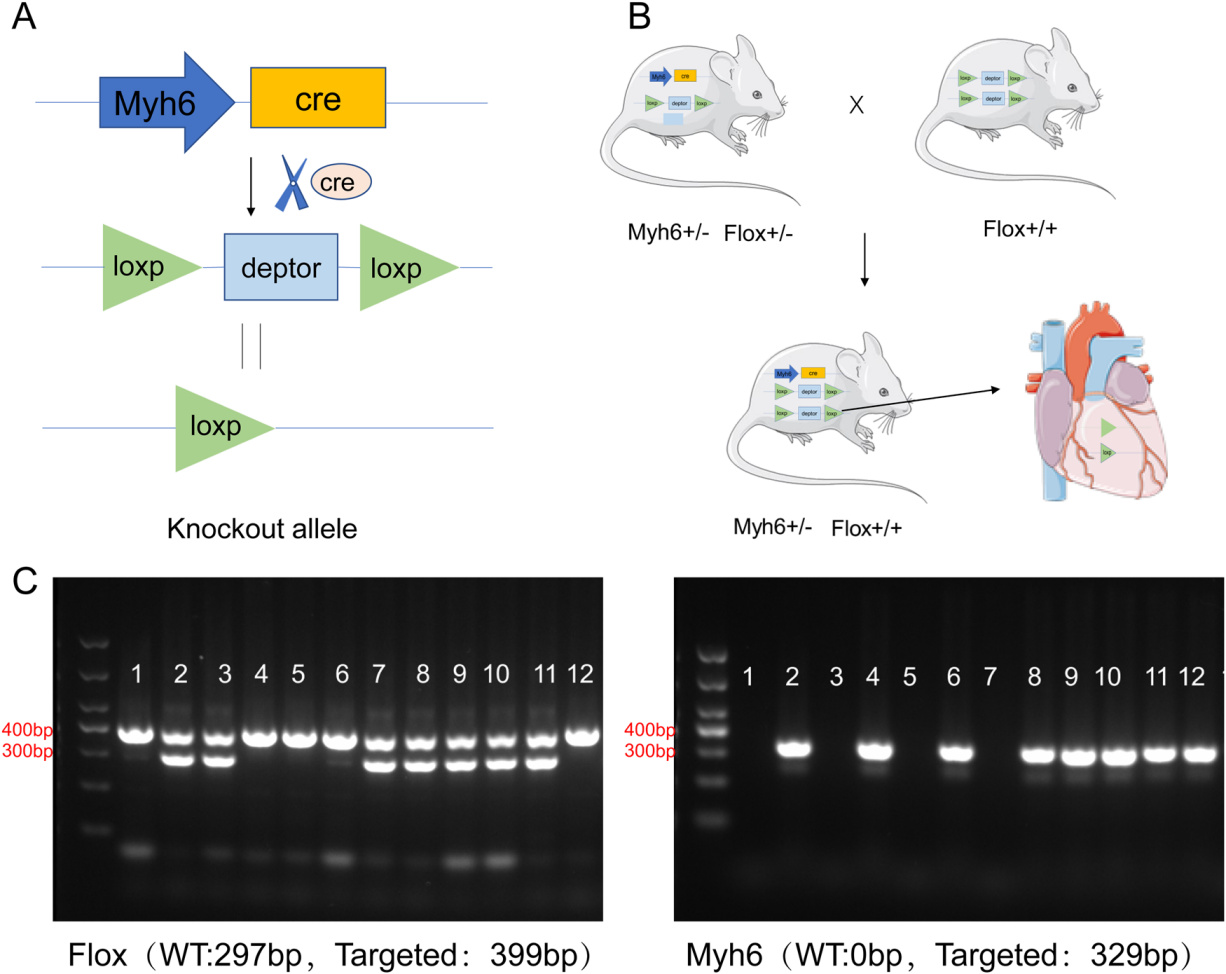
Generation of myocardial cell-specific Deptor knockout mice. **A**, **B** The schematic diagram represents the generation of the Deptor knockout mice. **C** RT-PCR was used to verify the locations of the genotyping primers and the results of genotyping.

To investigate the effects of Deptor knockout on myocardial I/R injury, we crossed Deptor Floxp3 mice with MyH6 Cre mice (see schematic diagram, Fig. 1A) to obtain the cardiomyocyte-specific Deptor knockout mice. FLOX and MYH6 genotyping were identified by RT-PCR (Fig. 1B, C). The heart from Fig. 1B was from https://smart.servier.com/smart_image/heart/(Non-commercial use citation).

### Deptor knockout aggravates myocardial I/R injury

**Fig. 2:**
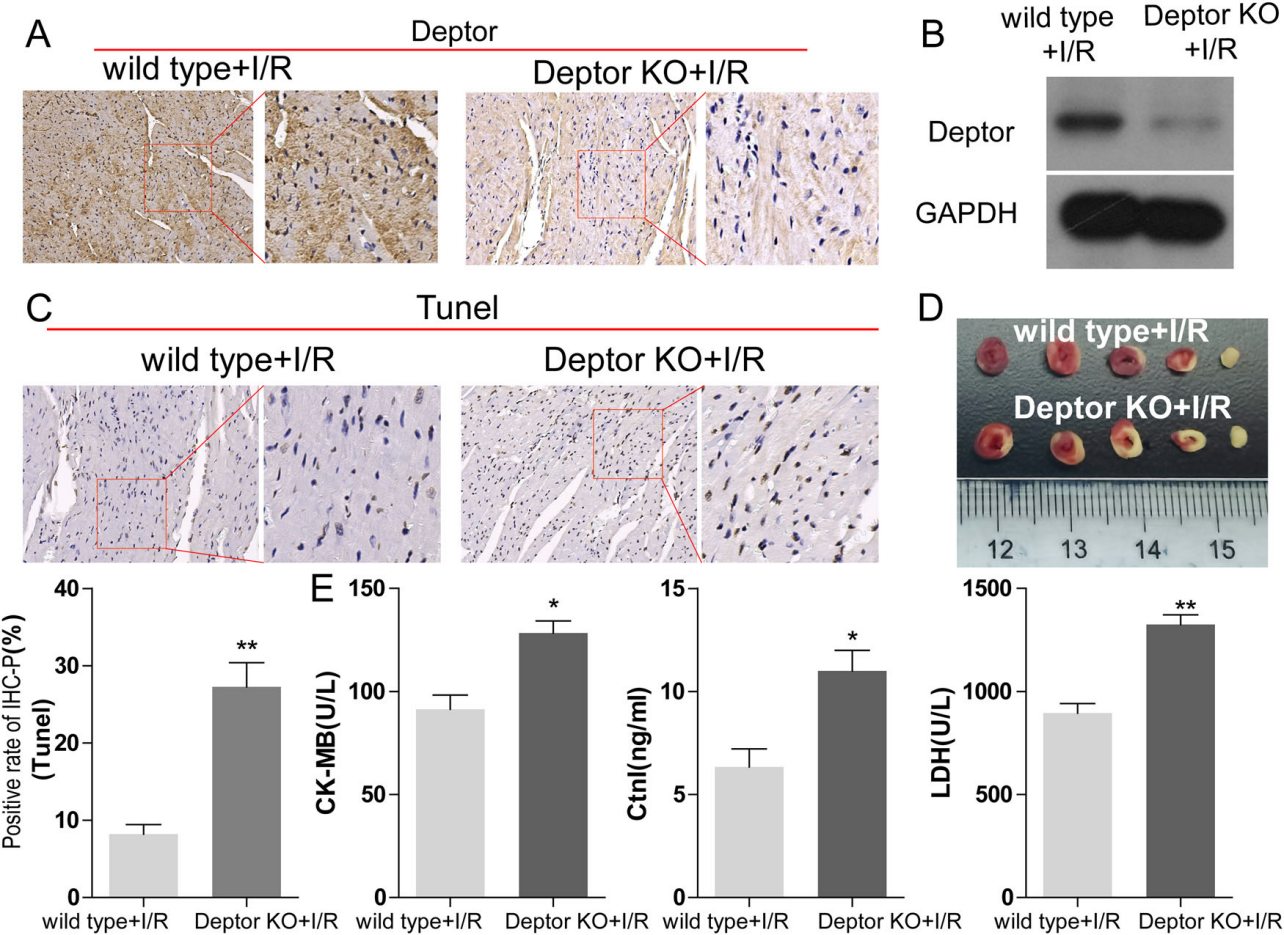
Myocardial cell Deptor knockout mice exhibit aggravated myocardial I/R injury. The expression of Deptor was determined using immunohistochemistry (**A**) and western blot (**B**). **C** TUNEL assay was used to determine myocardial cell apoptosis. **D** TTC staining analyzed the area of myocardial infarction (*n* = 5). **E** ELISA determined expression levels of CK-MB, CtnT, and LDH expression in I/R or Deptor KO+I/R. *P < 0.05, **P < 0.01 vs wild type+I/R.

Fresh cardiac tissue was collected from the Deptor KO+I/R group and the control mice of the same type (wild type+I/R) group, and Deptor expression was determined by western blot and immunohistochemistry. Deptor expression was significantly decreased in the Deptor KO+I/R group (Fig. 2A, B). TTC staining indicated that the infarction size and injury were increased in Deptor KO+I/R group (Fig. 2D). Evaluation of apoptosis by TUNEL assay demonstrated a significant increase in apoptotic cells in the Deptor KO+I/R group, and ELISA evaluation of CK-MB, LDH, and serum levels of CtnT/I enzyme indicated that the expression of CK-MB, LDH and CtnT/I were significantly higher in the Deptor KO+I/R group than the wild type+I/R group (Fig. 2C, E, respectively). These results suggest that Deptor knockout in myocardial cells aggravates myocardial I/R injury.

### The effects of Deptor knockout in cardiomyocytes on an in vitro I/R model

**Fig. 3:**
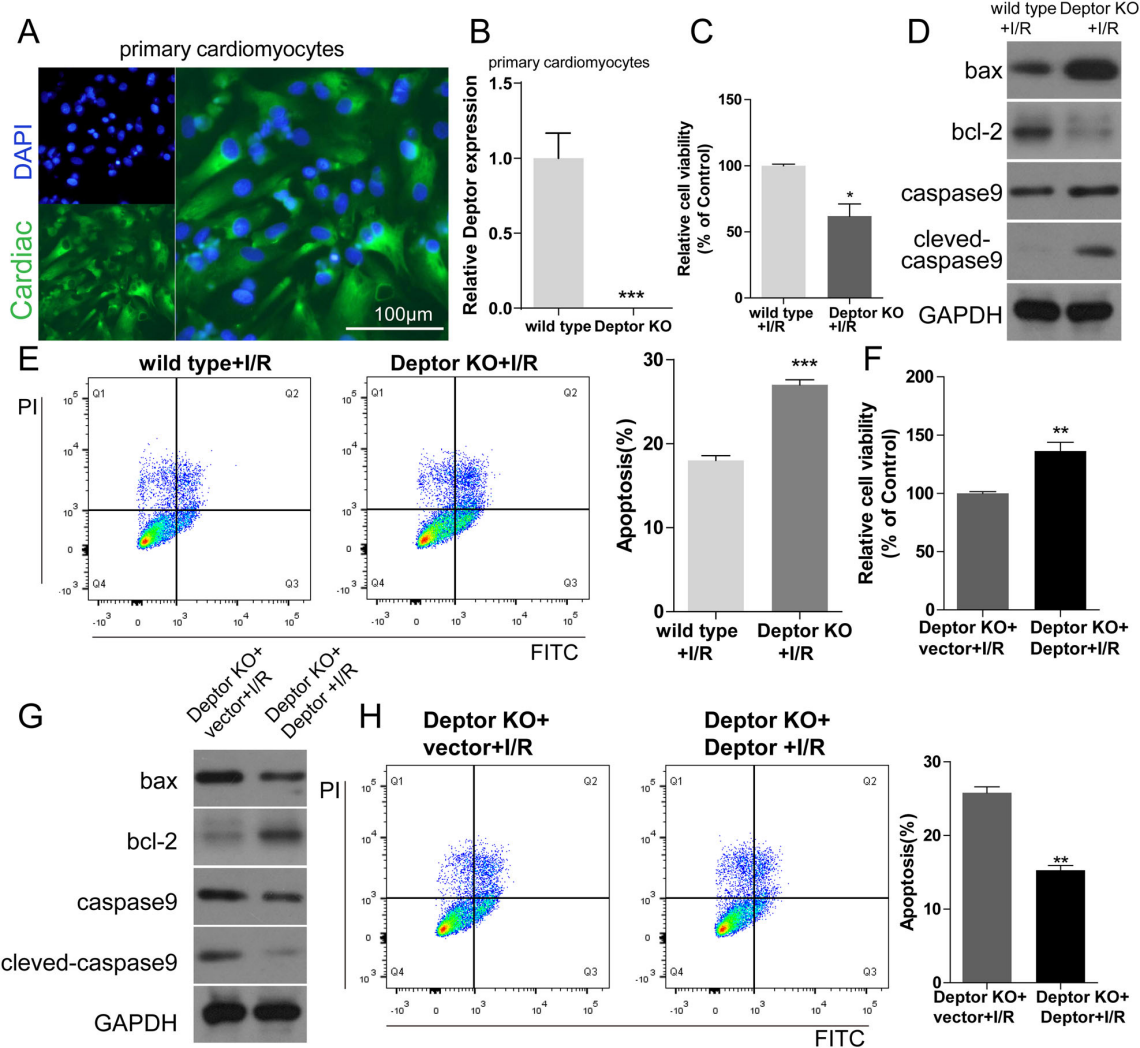
The effects of Deptor knockout in primary cardiomyocytes in an in vitro I/R model. **A** Immunofluorescence identification of expression of cardiac markers in primary cardiomyocytes from Deptor KO mice. **B** The expression of Deptor was determined using RT-qPCR. ***P < 0.001. **C** Cell viability in wild type and Deptor KO primary cardiomyocytes during I/R were examined using CCK-8 assay. *P < 0.05 vs. wild type+I/R. **D** Western blot analysis of apoptosis-related protein expression in wild type and Deptor KO primary cardiomyocytes. **E** Flow cytometric analysis of primary cardiomyocyte apoptosis. ***P < 0.001. **F–H** Cell viability, expression of apoptosis-related proteins, and the number of apoptotic primary cardiomyocytes in Deptor KO+vector and Deptor KO+Deptor during I/R were evaluated using CCK-8 assay, western blot, and flow cytometry, respectively. **P < 0.01 vs. wild type+I/R. **P < 0.01 vs. Deptor KO+vector +I/R.

**Fig. 4:**
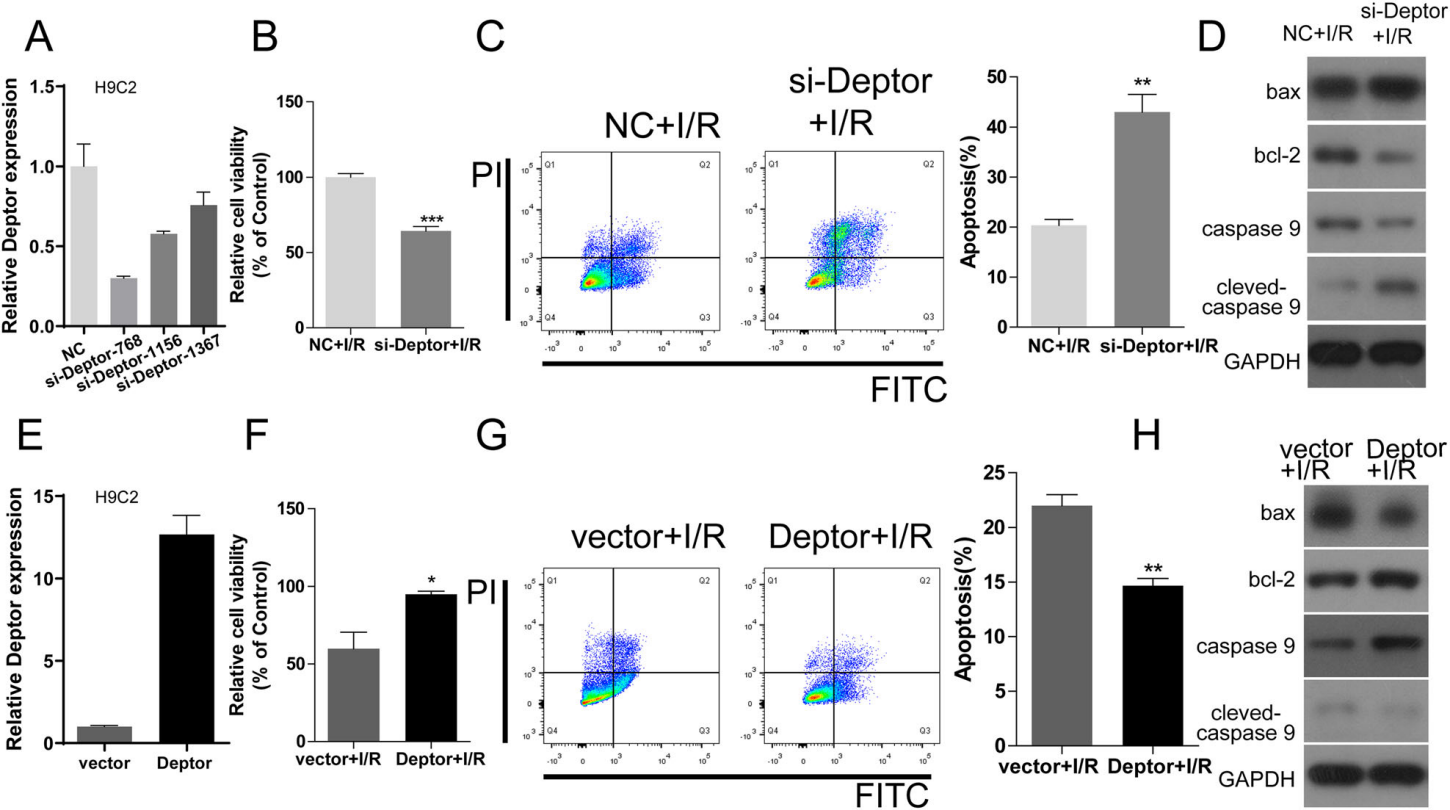
The effects of Deptor knockout in H9C2 cardiomyocyte cells in an in vitro I/R model. **A**, **E** The lever of Deptor was detected using RT-qPCR. **B**, **F** Cell viability was determined in NC+I/R, si-Deptor+I/R or vector+I/R and Deptor+I/R (H9C2 cells), ***P < 0.001 vs. NC+I/R; *P < 0.05 vs. vector +I/R, respectively. **C**, **G** The number of apoptotic H9C2 cells was determined in NC+I/R, si-Deptor+I/R or vector+I/R, and Deptor+I/R groups using flow cytometry. **P < 0.01 vs. NC+I/R; **P < 0.01 vs. vector+I/R respectively. **D**, **H** Western blot analysis of apoptosis-related proteins in NC+I/R, si-Deptor+ I/R, or vector+I/R and Deptor+I/R (H9C2 cells).

Immunofluorescence was used to confirm Deptor knockout in mouse primary myocardial cells (Fig. 3A). The expression of Deptor was determined after transfectedb with si-Deptor or Deptor plasmid (Fig. 3B and Fig. 4A, E). CCK-8 and flow cytometry were used to detect cell viability and apoptosis, respectively, in primary myocardial cells from Deptor KO mice and Deptor knockout H9C2 cell after I/R induction. Deptor knockout decreased cell viability and increased apoptosis, with wild type+IR or NC+I/R cells (Figs. 3C, E and 4B, C). Moreover, Deptor knockout also up-regulated the expression of Bax, caspase 9, and cleaved-caspase 9, and down-regulated Bcl-2 expression (Figs. 3D and 4D). In addition, transfection of a Deptor-overexpression plasmid into Deptor knockout primary cardiomyocytes or H9C2 cardiomyocytes increased cell viability reduced the number of apoptotic cells, and down-regulated the expression of Bax, caspase 9, and, cleaved-caspase 9, and up-regulated Bcl-2 expression (Fig. 3F, G and 4F–H). These results further confirm that Deptor knockout enhances the severity of myocardial I/R injury in vitro.

### Deptor protects against I/R myocardial injury by regulating the mTOR pathway and autophagy

**Fig. 5:**
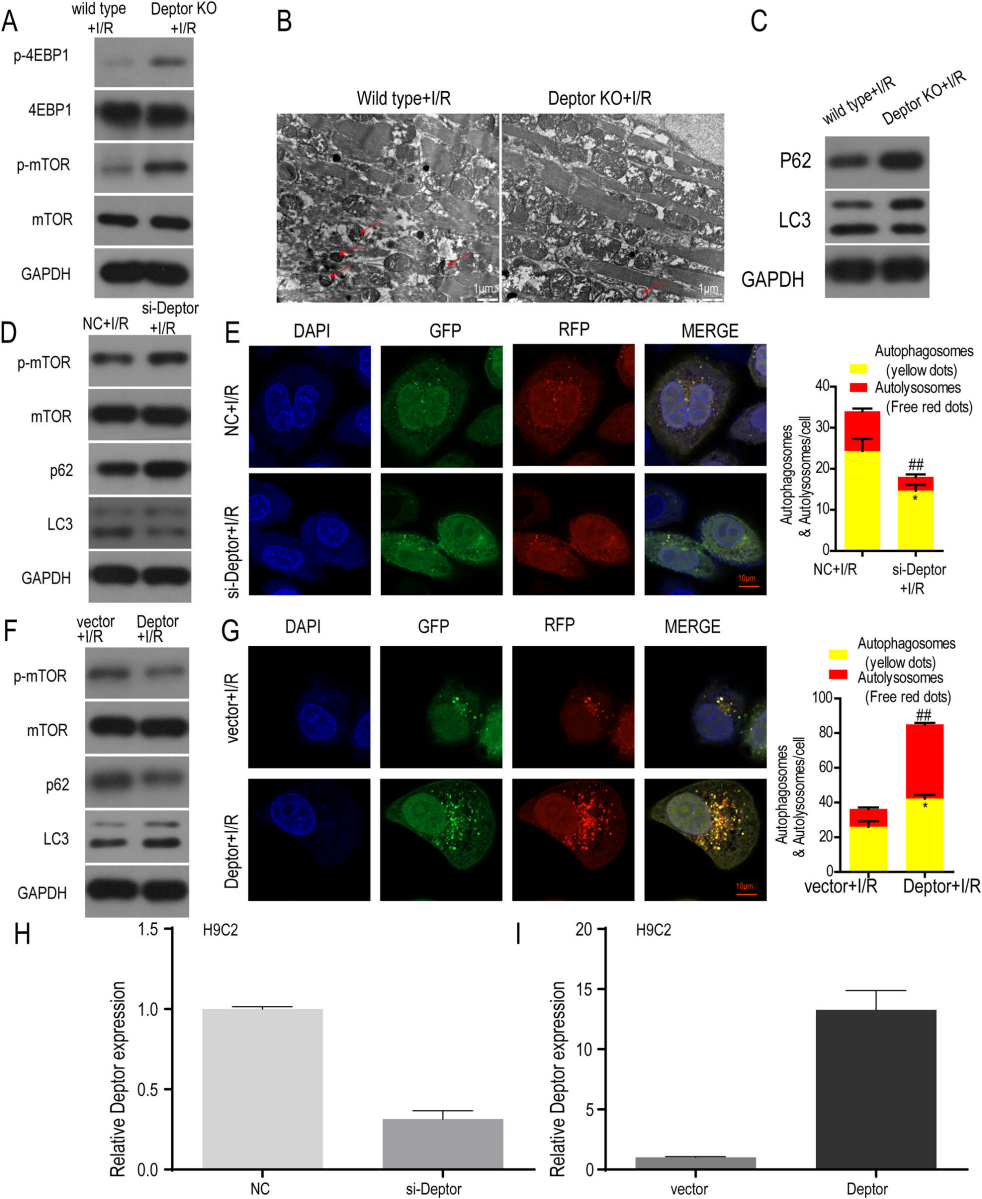
Deptor protects against I/R myocardial injury by regulating mTOR and autophagy pathways. **A** The expression of p-4EBP1, 4EBP1, p-mTOR, and mTOR was evaluated in the wild type and Deptor KO+I/R groups in vivo. **B** Transmission electron microscopy analyzed the number of autophagosomes in the wild type and Deptor KO+I/R groups in vivo. **C** Expression of autophagy-related proteins p62 and LC3 in wild type and Deptor KO+I/R groups from myocardium. **D**–**G** Western blot and confocal microscopic analysis of the expression of p-4EBP1, 4EBP1, p-mTOR, and mTOR protein and the expression of autophagy LC3 double fluorescence in NC+IR, si-Deptor+IR, vectro+IR, Deptor+IR (H9C2 cells). **H**, **I** The Deptor expression after transfected with si-Deptor or Deptor plasmid was dermined.

Given the regulatory role of Deptor on the mTOR pathway and autophagy, we hypothesized that Deptor could inhibit mTOR pathway activation and promote autophagy. Western blot analysis found that Deptor KO significantly up-regulated the expression of p-mTOR, p-4EBP1, and p62, and down-regulated the expression of LC3 in the myocardium (Fig. 5A, C). Transmission electron microscopy (TEM) analysis showed that the number of autophagosomes was decreased in the myocardium of Deptor KO specimens (Fig. 5B). Furthermore, Deptor KO up-regulated the expression of p-mTOR, p62, and down-regulated the expression of LC3; while rescue of Deptor expression with a Deptor-overexpression plasmid had the opposite effect on these proteins (Fig. 5D, F). Confocal microscopy also demonstrated that the LC3 autophagy double fluorescence was decreased following transfection with Deptor siRNA, and increased following transfection with Deptor expression plasmid (Fig. 5E, G, respectively). Treatment with si-Deptor could down-regulate Deptor expression, while Deptor plasmid transfection could up-regulate it (Fig. 5H, I).

### The mTOR pathway mediates the role of Deptor in promoting autophagy and protecting cardiomyocytes from I/R injury

**Fig. 6:**
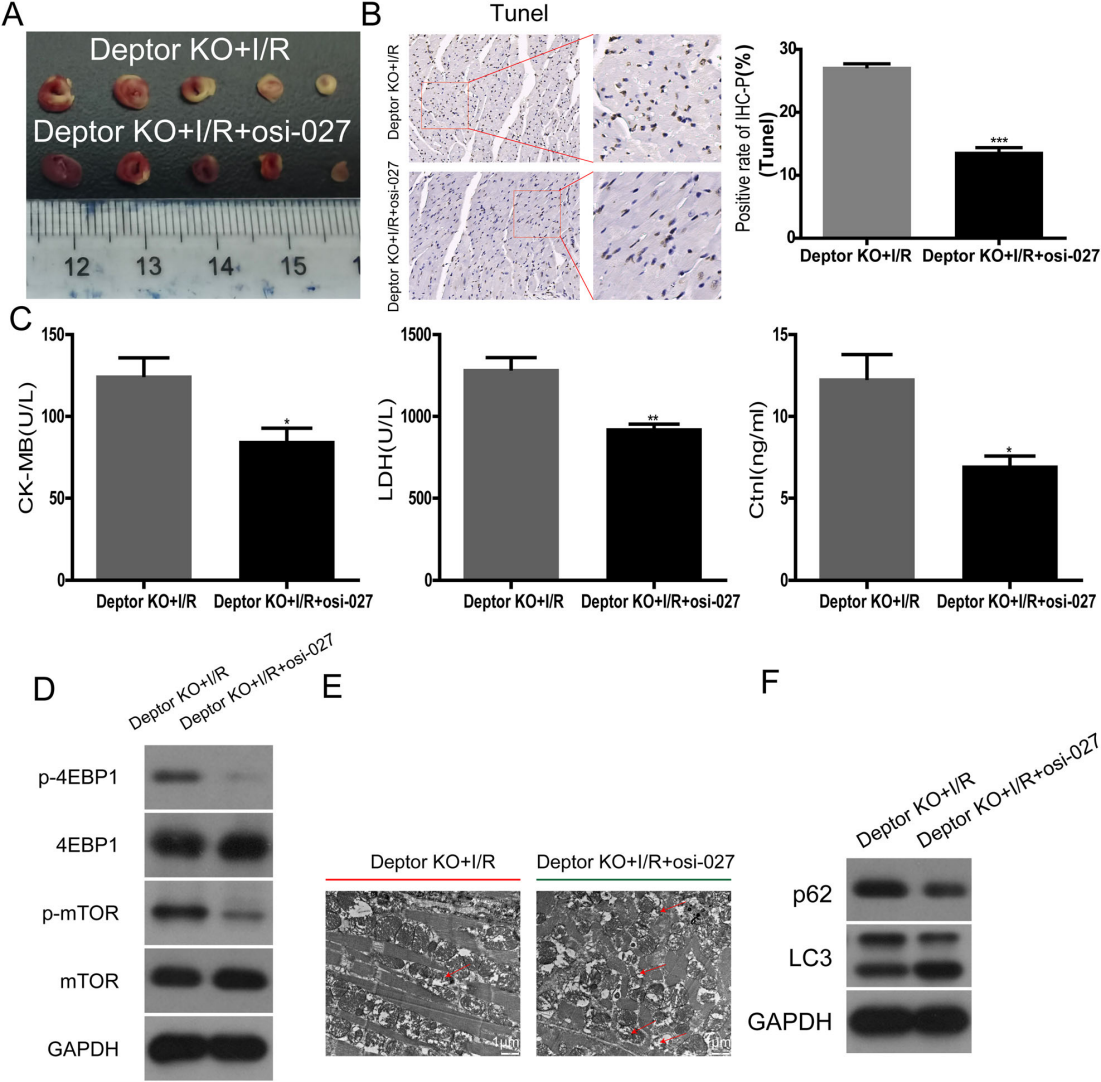
The mTOR pathway mediates the role of Deptor in promoting autophagy in vivo. **A** TTC staining analysis of the area of myocardial infarction in Deptor KO+I/R and Deptor KO+I/R+ following treatment with OSI027 (*n* = 5). **B** TUNEL determination of the rate of myocardial cell apoptosis. **C** ELISA determination of expression of CK-MB, CtnT, and LDH expression after treatment with OSI027 combined with Deptor KO under I/R conditions. *P < 0.05, **P < 0.01, vs. Deptor KO+I/R. **D** The level of p-4EBP1, 4EBP1, p-mTOR, and mTOR determined in Deptor KO+I/R and Deptor KO+I/R+OSI027 groups. **E** Transmission electron microscopy analysis of the number of autophagosomes in Deptor KO+I/R and Deptor KO+I/R+OSI027 groups **F** Western blot evaluation of p62 and LC3 in Deptor KO+I/R and Deptor KO+I/R+OSI027 groups.

**Fig. 7:**
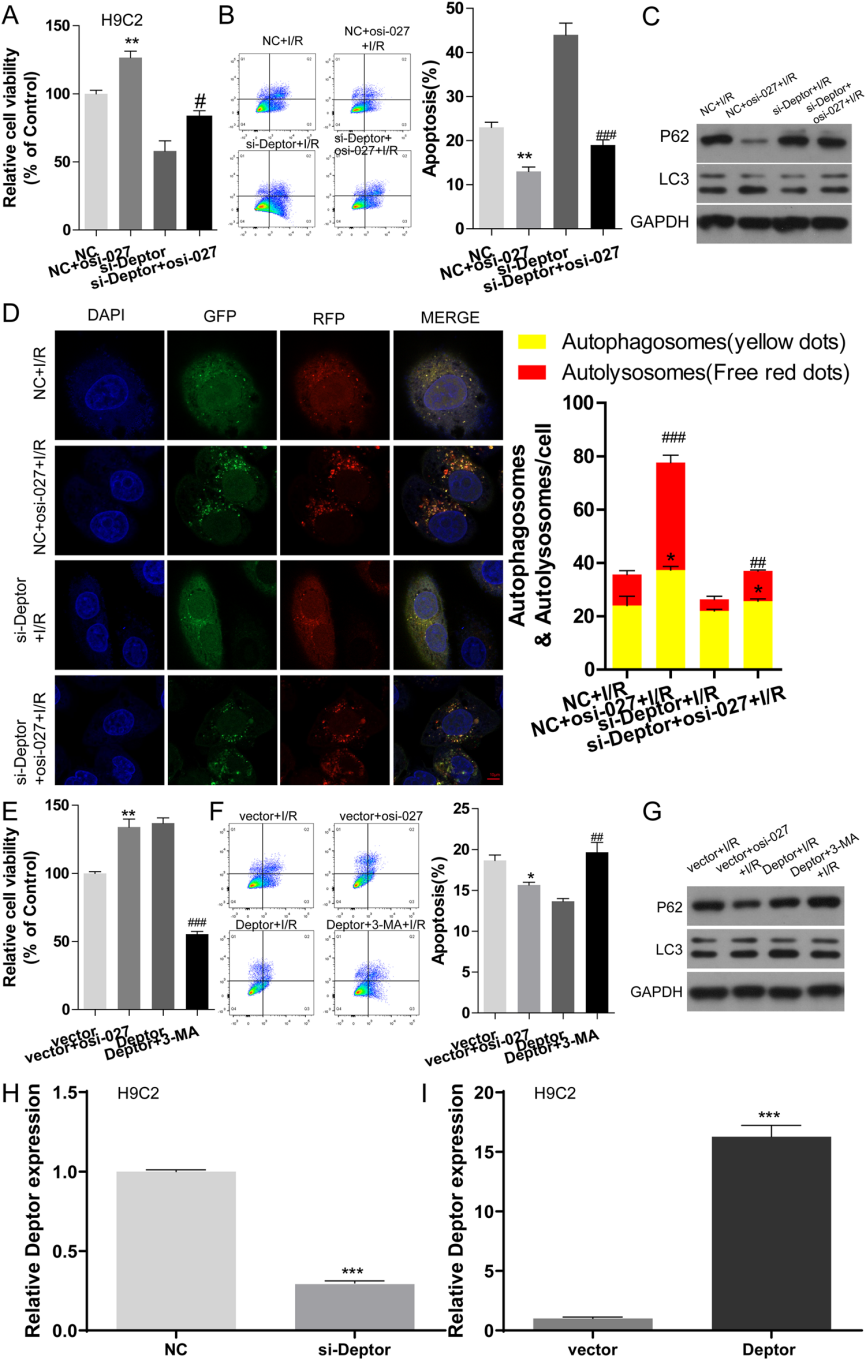
The role of Deptor in promoting autophagy and protecting I/R cardiomyocytes injury in vitro*.* **A**–**C** Cell viability, the number of apoptotic H9C2 cells, and autophagy-related protein expression in different treatment groups were evaluated using CCK-8 analysis, flow cytometry, and western blot, respectively. **P < 0.01 vs. NC; # < 0.05, ^##^*P* < 0.01 vs. si-Deptor. **D** Confocal microscopy examination of autophagy LC3 double fluorescence following H9C2 cells treatment with OSI027 or si-Deptor alone, or combination of OSI027 and si-Deptor under I/R. *P < 0.05 vs. NC+I/R (Autophagosones, yellow); ^##^*P* < 0.01, ### < 0.001 vs NC+I/R(Autolysosomes, red). **E**–**G** CCK-8 analysis, flow cytometry, and western blot were used to detect H9C2 cell viability, the rate of apoptotic H9C2 cells, and the expression of autophagy-related proteins in H9C2 cells. *P < 0.05, **P < 0.01 vs. vector; # < 0.05, ^##^*P* < 0.01 vs. si-Deptor. **H**, **I** The expression was determined using RT-qPCR. ***P < 0.001.

OSI027, a common inhibitor of mTORC1 and mTORC2, was used to inhibit mTOR pathway in Deptor KO I/R models in vivo and in vitro. TTC staining revealed a significant decrease in infarction size of Deptor KO+I/R+OSI027 (Fig. 6A). Compared with Deptor KO+I/R, the number of apoptotic cardiomyocytes was also significantly reduced in the Deptor KO+I/R+OSI027 group, as indicated by TUNEL analysis (Fig. 6B). ELISA results showed that the expression of cK-MB, LDH, and CtnT/I were significantly down-regulated in the Deptor KO+I/R+OSI027 group (Fig. 6C). OSI027 treatment of Deptor KO+I/R models down-regulated the expression of p-mTOR, p-4EBP1, and p62, up-regulated the expression of LC3, and increased the number of autophagosomes (Fig. 6D–F). CCK-8 and flow cytometry indicated that Deptor siRNA combined with OSI027 could increase cell viability and apoptosis, in comparison with Deptor siRNA treatment alone (Fig. 7A, B). WB and confocal microscopy showed that OSI027 could reverse the downregulation of LC3 by Deptor siRNA (Fig. 7C, D). Moreover, the protective effects of Deptor expression by Deptor-overexpression plasmid on myocardial I/R injury were blocked or partially blocked by the autophagy inhibitor 3-MA (Fig. 7E–G). The interference efficiency of si-Deptor or Deptor plasmid was determined using RT-qPCR (Fig. 7H, I).

## Discussion

Myh6 (also known as αMHC, Myhc, and Myhca) encodes the cardiac muscle-specific protein, alpha-myosin heavy chain, which is dynamically expressed in cardiomyocytes and is of great significance to cardiac development. MYH6 is expressed at low levels in adult human cardiomyocytes. Myh6-Cre is a useful genetic tool used to delete a target gene in cardiomyocytes [26]. In this present study, we constructed cardiomyocyte-specific Deptor knockdown mice. Deptor KO enhanced the size of myocardial infarction and increased apoptosis in myocardial cells in an I/R model. Deptor is an mTOR-binding molecule that binds not only mTORC1, but also mTORC2. Deptor is an mTOR inhibitor, and inhibits mTORC1/2 activity by binding to mTORC1/2 through its PDZ domain. Deptor can also be phosphorylated by mTORC1 or p70S6K. Knockdown of Deptor increased p-mTOR and p-4EBP1 expression, and overexpression of Deptor reduced p-mTOR and p-4EBP1 expression.

Autophagy is a tightly regulated, multi-step, dynamically balanced process that leads to degradation of subcellular components by the formation of autophagocytes that fuse with lysosomes and produce autophagolysosomes. Autophagy is activated in response to nutritional deficiency or metabolic stress, and maintains tissue homeostasis by recycling dysfunctional proteins and organelles [27, 28]. One key biological marker of autophagy is LC3, which makes up the autophagosome membrane. During autophagy, cytoplasmic LC3-1 binds with phosphatidylethanolamine to form the LC3-II complex, which is then incorporated into the autophagosome membrane. Another widely accepted marker of autophagic flux is the autophagic receptor SQSTM1 and p62, which connect the autophagic substrates to the autophagic membrane [29]. Autophagy dysfunction is associated with a variety of pathologic diseases, including cancer, neurodegenerative diseases, and cardiovascular diseases [7]. Autophagy of cardiomyocytes plays an important role in cardiovascular diseases and autophagy is a major regulator of cardiac homeostasis and function [8]. Ischemia-induced autophagy inhibits myocardial remodeling, improves myocardial function during AMI, and can be protective for myocardial cells [30, 31]. The enhancement of upstream expression of autophagy-related genes or the blocking of downstream degradation leads to the accumulation of p62, which is eventually incorporated into the mature autophagosome and degraded during autophagy. Therefore, the levels of p62 are negatively correlated with autophagy activity [32]. In addition, the levels of p62 are often combined with the ratio of LCII/LCI to evaluate the level of autophagy. During autophagy, p62 binds to ubiquitinated proteins and forms a complex with LC3-II protein located on the autophagosome inner membrane, which is then degraded in the autophagic lysozyme [33, 34]. Therefore, when autophagy occurs, p62 protein is continuously degraded in the cytoplasm. When autophagy activity is weakened and autophagy is deficient, p62 protein will accumulate continuously in the cytoplasm. p62 is one of the marker proteins reflecting autophagy activity, and its levels indirectly reflect the clearance of autophagosomes [19]. In our in vivo and in vitro models of I/R, Deptor knockout in cardiomyocytes inhibits autophagy by regulating the expression of p62 and down-regulating the expression of LC3. The initiation of autophagy is regulated by Class I and Class III PI3K. Class I PI3K indirectly inhibits autophagy through AKT and mTOR, and Class III PI3K directly promotes autophagy through interaction with Beclin1 [18]. Beclin1 and mTOR, two important autophagy-related proteins, play an important role in myocardial I/R injury [11, 12]. mTOR regulates autophagy during ischemia through the AMPK-mTOR pathway and PI3K-Akt-mTOR pathway, and Beclin1 also plays an important role in I/R [13]. In concordance, our data demonstrated that Deptor regulates changes in autophagy, as seen by the increase in phosphorylated mTOR levels following transfection with Deptor siRNA, thereby inhibiting autophagy, while overexpression of Deptor in H9C2 cardiomyocytes increased autophagy by inhibiting the mTOR pathway.

## Conclusion

In conclusion, this study confirmed that Deptor inhibits cardiomyocyte autophagy by activating the mTOR pathway, thereby aggravating I/R injury in cardiomyocytes. The results provide justification for pursuing Deptor as a novel target to improve the protective effect of autophagy during myocardial I/R injury.

## Materials and methods

### Generation of cardiomyocyte-specific Deptor knockout mouse model

Deptor flox/flox mice were generated by Gempharmatech Co., Ltd. Deptor flox/flox mice were bred with myh6-cre mice to obtain cardiomyocyte-specific knockout Deptor mice (***myh6-cre*** Deptor flox/flox mice; Deptor KO mice).

### Genotyping identification

Genomic DNA was extracted from the tails of mice. Harvested tissues were incubated with proteinase K for 12 h at 55 °C and then centrifuged for 8 min. Isopropanol was added to the collected supernatant to precipitate DNA. Next, PCR amplification was performed according to the following reaction parameters: 95 °C 5 min; 95 °C 30 s, 65 °C 30 s, 72 °C 45 s, 20 cycles; 95 °C 30 s, 55 °C 30 s, 72 °C 45 s, 20 cycles; 72 °C 5 min; 10 °C hold. The genotyping primer sequences are as follows:

Myh6-Cre mouse line:

5′-CCTGCTGTCCATTCCTTATTCCATA-3′

5′-ATATCCCCTTGTTCCCTTTCTGC-3′

Flox mouse line:5′-GTTCTACATCTTCATCTCCAGGC-3′

5′-CACCCAAGTAGAGGCACTTACTG-3′

### Establishment of the myocardial I/R model

The myocardial I/R model was performed as previously described [35]. Briefly, we anesthetized mice with intraperitoneal injection of 3% pentobarbital sodium, and we established the I/R injury model using the coronary artery ligation (LAD) method. We opened the left thoracic cavity to expose the heart and ligated around the LAD with a 6-0 ligature for 30 min, followed by reperfusion for 120 min. The mice were sedated with 1% sodium pentobarbital. The mice were euthanized by intraperitoneal injection of 2% isoflurane 2 weeks after the I/R operation. Finally, we harvested heart tissue and sliced into 1 mm segments.

### Immunohistochemical staining

Myocardial tissues were obtained from mice and fixed in formalin. The fixed tissues were embedded in paraffin blocks and cut into 4 μm-thick sections. After mounting on slides, immunohistochemistry (IHC) was performed. Sections were stained with primary antibodies to Deptor (1:200, Cell Signaling Technology) and Deptor expression was observed by HRP-DAB immunostaining.

### TUNEL assay

TUNEL staining was used to determine myocardial cell apoptosis. The myocardium from the apex of the left ventricle was cut into small pieces, fixed with paraformaldehyde phosphoric acid, dehydrated with gradient ethanol, made transparent with xylene, and embedded with paraffin. The thickness of the sections was 5 μm, and sections were stained according to the TUNEL kit(Roche, Basel, Switzerland). Positive staining, indicated by brown-yellow particles of the nuclei, was observed under a microscope.

### TTC staining

The area of myocardial infarction was determined by TTC staining area. The heart was removed quickly at the end of perfusion, and frozen for 30 min at −20 °C. Next, the heart tissue was cut into 1–2 mm thick slices from the apex to the bottom of the heart. The myocardial slices were incubated with 1%TTC phosphate buffer solution at 37 °C for 30 min. The surviving myocardium was colored brick red, while the infarcted myocardium was colored grayish white. The two kinds of myocardial tissues were fixed with 10% formalin solution, and weighed to calculate the ratio of myocardial infarction area to total myocardial weight. Myocardial infarction area (%) = (myocardial infarction area/total myocardial weight) × 100%.

### ELISA assay

The expression of myosin and creatine kinase isoenzyme (CK-MB) was detected by ELISA, and the expression of cardiac troponin (cTnT/I) troponin I was determined by a cTnT Detection Kit, according to manufacturers’ instructions.

### Cytotoxicity LDH analysis

A Cytotoxicity LDH Assay Kit-WST (Dojindo, Kumamoto, Japan) was used to determine cytotoxicity based by LDH released by damaged cells, according to manufacturer’s instructions.

### Isolation of primary myocardial cells

The hearts of mature C57BL/6J mice were digested with a mixture of trypsin and collagenase (0.4% collagenase: 0.05% trypsin = 2:1) at 37 °C until the color of the myocardium turned from red to white. The cell suspension was placed in the incubator for one and a half hours, and a differential adherent cell separation technique was used to remove most of the fibrous cells. Then, BrdU was used to interfere with mitosis of the cells and to inhibit the growth of fibroblasts. More than 90% of the cardiomyocytes were obtained by continuous culture. Finally, the growth and morphology of cardiomyocytes from day 1 to 7 were observed, and the spontaneous pulsation frequency was recorded.

### Western blot analysis

Myocardial cells were collected, lysed, and protein was extracted. Briefly, 40 μg protein was separated using 10% SDS-PAGE and then transferred to a PVDF membrane (Millipore). The membranes were blocked with TBST for 2 h at 37 °C and incubated overnight with primary antibodies (Deptor, Bax, Bcl-2, Caspase9 Cleved-casapase9, p62, 4EBP1, mTOR, diluted 1:1000 in TBS-T) at 4 °C, and then incubated with corresponding HRP-conjugated secondary antibodies for 2 h at 37 °C. GADPH was used as an internal control. The protein bands were visualized using chemiluminescence (GE Healthcare, Piscataway, NJ, USA).

### Immunofluorescence staining

Primary myocardial cells were seeded into 48-well plates at 3 × 10^3^ cells/well, fixed with 4% formaldehyde for 15 min, blocked with 5% BSA for 30 min, and incubated with anti-Cardiac Troponin I antibody (1:200) (Abcam, Cambridge, USA) at 4 °C overnight, later the cells were incubated with FITC-conjugated secondary antibody (Abcam) at 4 °C for 2 h. Nuclear staining was performed with DAPI (Sigma) for 2 min. The stained cells were observed using an inverted fluorescence microscope (Olympus, Tokyo, Japan).

### Cell viability

Primary myocardial cells of Deptor KO mice and Deptor knockout H9C2 cells were seeded into 96-well plates at a density of 3 × 10^3^ cells/well. Cell viability was examined with a cell counting kit-8 assay (CCK-8; Dojindo). Briefly, 10 μL CCK-8 solution was added into well and incubated for 3 h. An MRX II microplate reader (Dynex Technologies, Chantilly, USA) was used to determine the absorbance at 450 nm.

### Transfections

H9C2 cells were seeded into 24-well plates at a density of 1 × 10^5^ cells/well and cultured overnight prior to transfection. The cells were then transfected with negative siRNA, Deptor siRNA, vector, or vector Deptor (GenePharma, Shanghai, China) using Lipofectamine 2000 (Invitrogen; Carlsbad, CA, USA) according to the manufacturer’s protocol for 6 h. The Deptor siRNA primer sequences used are as follows:

Deptor-rat-768

Forward primer: 5′-GCACAGCAUCAUACAGCAUTT-3′

Reverse primer: 5′-AUGCUGUAUGAUGCUGUGCTT-3′

Deptor-rat-1156

Forward primer: 5′-CCAUAUGCAAGGAAGACAUTT-3′

Reverse primer: 5′-AUGUCUUCCUUGCAUAUGGTT-3′

Deptor-rat-1367

Forward primer: 5′-GCCCGAGGACAAUUGUCAUTT-3′

Reverse primer: 5′-AUGACAAUUGUCCUCGGGCTT-3′

### Quantitative reverse transcription PCR (RT-qPCR)

Total RNA from H9C2 cells was extracted using TRIzol reagent (Invitrogen, Carlsbad, CA, USA) according to the manufacturer’s instructions. Total RNA was reverse transcribed using a PrimeScript RT Reagent Kit with a gDNA Eraser (Takara, Dalian, China; RR047A). After the reverse transcription reaction, 1 μL of complementary DNA was used for subsequent qPCR reactions (SYBR Green dyestuff, TaKaRa Biotechnology, Dalian, China) in an ABI 7500 real-time PCR system (Applied Biosystems, Foster City, CA, USA) according to the manufacturer’s instructions. U6 gene was used as an internal control. The relative levels of TUG1 expression were assessed using the comparative 2^−ΔΔCt^ method [36]. The following primer sequences for TUG1 were used:

rno-Deptor:

Forward primer: 5′-GAAGTAAGCCATGCCACATCCA-3′;

Reverse primer: 5′-CGTTGACAGAGACGACAAACTGAC-3′.

### Flow cytometry

Flow cytometry confirmed the rate of apoptosis cells. Cells were treated with a trypsin-EDTA (0.25%) solution and centrifuged at 1000 × *g* for 5 min. Cells were washed with PBS three times, and an Annexin V-FITC/PI (BD) antibody was used to stain apoptotic cells, according to the manufacturer’s instructions.

### Transmission electron microscopy

TEM was used to detect the formation of autophagosomes. After cardiac perfusion, left ventricle was cut into small pieces and fixed in 2.5% glutaraldehyde and 2% osmium for 2 h. Left ventricle tissue was then dehydrated with increasing concentrations of ethanol (50%, 70%, 80%, and 90%) for 15 min, then dehydrated with 100% ethanol three times for 20 min. Next, the ethanol was replaced with acetone for 15 min, twice in total. The samples were placed in pure embedding agent and embedded in a plate at 65 °C for 48 h. Samples were prepared for TEM by staining with uranium acetate and lead citrate. The formation of autophagosomes was observed by TEM.

### Confocal microscopy

Myocardial cells were transfected with adenovirus containing mRFP-GFP-LC3 construct. Laser confocal microscopy was used to detect of green (GFP) or red (mRFP) fluorescence protein expression. Yellow dots and red dots in the combined images represent autophagosomes and autolysosomes, respectively. The percentage increase in red dots in the combined image indicates the autophagy flux.

### Statistical analysis

All statistical analyses were performed using GraphPad Prism software 8.0 (La Jolla, CA, USA). The data are expressed as the mean ± standard deviation (SD) and analyzed by one-way analysis of variance followed by Tukey’s tests. A *p* value of *P < 0.05 was considered to indicate a significant difference.

## Supplementary information

Supplementary WB original

## Data Availability

We declare that all data support the conclusions of the study and all raw data will be made available upon request.
