# Supplementary material for: Deptor protects against myocardial ischemia-reperfusion injury by regulating the mTOR signaling and autophagy
Source: Cell Death Discov. 2024 Dec 19;10:508. doi: 10.1038/s41420-024-02263-1 (PMC11659626; doi:10.1038/s41420-024-02263-1)

Figure 2

Deptor


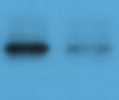


GAPDH


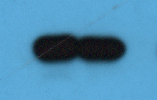


Figure 3

C

Bax


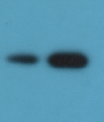


Bcl-2


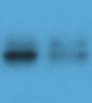


Caspase9


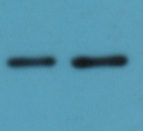


Cleved-caspase9


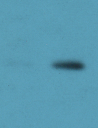


GAPDH


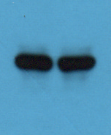


F.

Bax


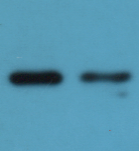


Bcl-2


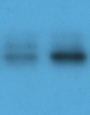


Caspase9


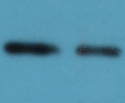


Cleved-caspase9


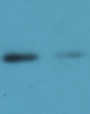


GAPDH


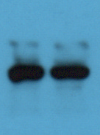


Figure 4

C

Bax


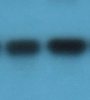


Bcl-2


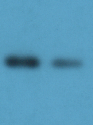


Caspase9


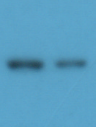


Cleved-caspase9


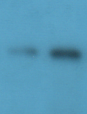


GAPDH


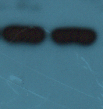


F.

Bax


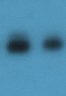


Bcl-2


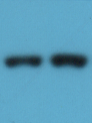


Caspase9


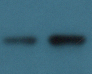


Cleved-caspase9


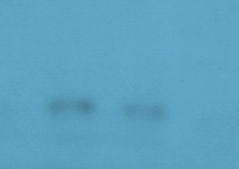


GAPDH


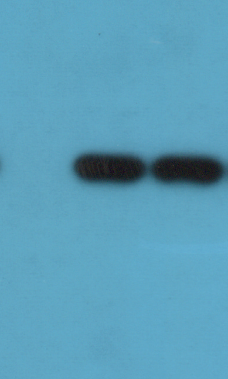


Figure 5

A

p-4EBP1


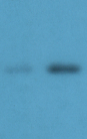


4EBP1


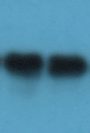


p-mTOR


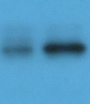


mTOR


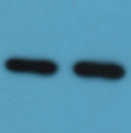


GAPDH


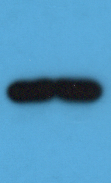


C

P62


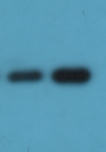


LC3


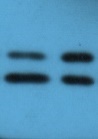


GAPDH


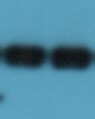


D

p-mTOR


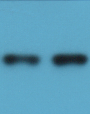


mTOR


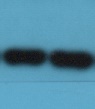


P62


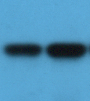


LC3


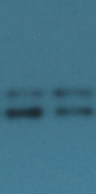


GAPDH


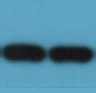


F

p-mTOR


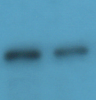


mTOR


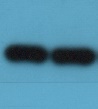


P62


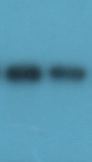


LC3


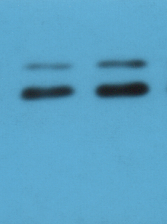


GAPDH


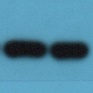


Figure 6

D

p-4EBP1


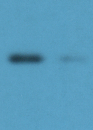


4EBP1


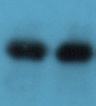


p-mTOR


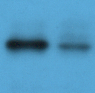


mTOR


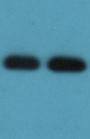


GAPDH


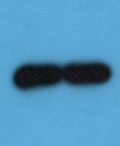


F.

P62


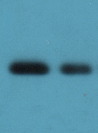


LC3


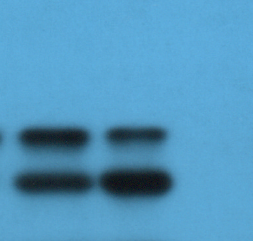


GAPDH


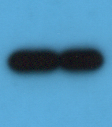


Figure 7

C

P62


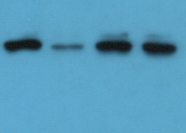


LC3


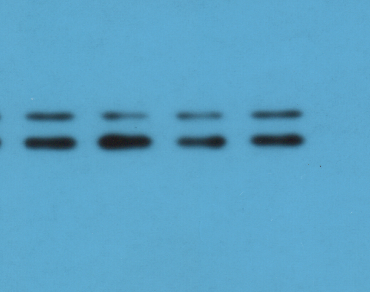


GAPDH


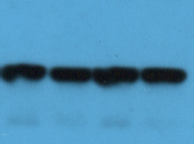


G

P62


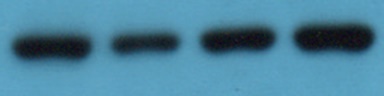


LC3


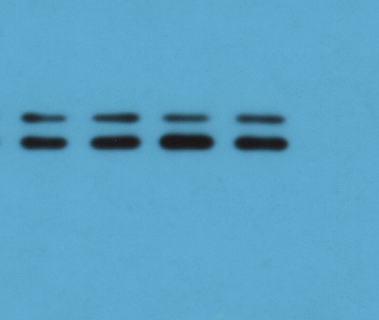


GAPDH


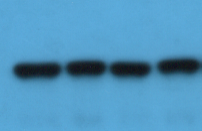

Supplement: Supplementary file 1 — Supplementary WB original [file 41420_2024_2263_MOESM1_ESM.docx]
